# Physiological Factors as Related to the Peridental Membrane, Cementum and Bone in Tooth Movement

**Published:** 1919-12

**Authors:** Martin Dewey


### PHYSIOLOGICAL FACTORS AS RELATED TO THE PERIDENTAL MEMBRANE, CEMENTUM AND BONE IN TOOTH MOVEMENT

BY MARTIN DEWEY, D.D.S.

In considering the physiology of the supporting structures of the tooth which may be said to consist of the alveolar process, the peridental membrane and the cementum, we can best arrive at those functions by considering briefly some of the embryological factors concerned in the development of those parts. The alveolar process belongs to that group of bones which are known as intramembranous bones, because they are primarily laid down within the mesoblastic embryonic tissue. The alveolar process has been developed for the sole purpose of supporting the tooth and consequently is subservient to the wish of the tooth. Wherever the tooth erupts, the alveolar process develops to support that tooth as a result of the mechanical stimulation produced by the development of the tooth and such other forces as are brought to bear upon it during the life of the individual.

In the individual, the alveolar process is composed of a compact plate covered with periosteum, which forms the labial and lingual portion of the process and a compact plate which lines the socket of the tooth which is covered by the peridental membrane. In between these plates we have a cancellous arrangement of bone, the spaces of which are filled with medullary tissue, otherwise known as bone marrow, which consists of various types of cells, the most important of which is the mesoblastic embryonic cells and osteoblasts, which are concerned in the future development of the bone. In considering the physiology of the alveolar process, we have to remember that the alveolar process develops and is repaired by the action of the osteoblasts which are found in the lacuna of the bone and in the medullary spaces. We have to discard the old theory that the peridental membrane and the periosteum had an osteogenetic function and were capable of repairing bone. All bone development comes from pre-existing osteoblasts or from mesoblastic embryonic cells and any repair or change which occurs as a result of tooth movement occurs as the result of these cells acting in conjunction with the osteoclasts and round cells which produce bone absorption. In pathologic conditions where there has been an absorption of the alveolar process, such a repair of the process as is obtained will be from the action of the osteoblasts in the adjoining healthy alveolar process and not from any osteogenetic function of the peridental membrane or periosteum. We may truly consider that the osteoblasts rush out into the pocket produced by the pathological condition and therein deposit bone which carries on repair produced by the immediate action of those cells. The function of the peridental membrane is wholly that of a physical function so far as the alveolar process and cementum is concerned. The peridental membrane is a thin, inelastic connective tissue membrane which forms the medium of attachment between the alveolar process and the cementum of the tooth. The attachment of the peridental membrane to the alveolar process is produced by the osteoblasts developing bone around the pre-existing fibers and binding it into the bone, very similar to the manner in which reinforced concrete rods are fastened into a cement wall. On the cemental side the same action takes place because the cementum develops from the basalar layer, and the cement lacuna lay down a layer of cementum on the dentin and thereby catch the ends of the fibers which are in apposition to the dentin. The peridental membrane has been developed from the connective tissue fibers which made up what in embryonic life is known as the dental sac, and these fibers have been increased as the result of physiological stress brought to bear on the crown of the tooth after the tooth had erupted.

The physiology of the cementum is purely that of a means of attachment between the fibers of the peridental membrane and the dentin. The cementum develops as an independent calcified tissue laid down on the dentin, and in no way is the cemental forming power dependent either upon the dentin or the peridental membrane. The older text books contain the statement that cementum was developed from the peridental membrane, or from cementoblasts with the natural habitants of the peridental membrane covering the roots of the tooth. Later investigations have failed to substantiate that theory, and by careful study we are forced to admit that there is no evidence to substantiate the belief that the peridental membrane is in any way concerned with the development of the cementum. From an examination of the cementum in various stages of development, and from a study of cross sections of the teeth in adult individuals everything points to the fact that the cementum is an independent calcified tissue, just as separate and distinct from the peridental membrane as bone is separate and distinct from the periosteum.

Repair of the cementum therefore occurs the same as the repair of bone, namely, by the escape of the cementoblasts from pre-existing cementum, thereby forming a new layer of cementum, the same as bone is formed in repair of fractures. From our present knowledge we can state that the alveolar process and cementum both are subject to extensive repair from injury, providing the pathologic conditions can be removed. A large amount of clinical evidence has been collected to show that large pockets in the alveolar process of roots of teeth will be refilled with alveolar process and repaired by cementum, providing the parts are healthy. Some of the most beautiful specimens of the development of the alveolar process which I have ever seen were specimens which were furnished me by Dr. Frank C. Pague, of San Francisco.

In closing, we will state that the large amount of evidence collected from the histological and clinical standpoint at the present time points very strongly to the fact that the physiology of the alveolar process is such as is very favorable for extensive repairs, both from pathologic conditions and traumatic injuries, and will respond to the proper application of force in the movement of teeth.

The peridental membrane is simply a physical structure holding the tooth in contact with the alveolar process and the gum tissue. This second function of the peridental membrane in holding the gingival tissue tight around the neck of the tooth is one which must be very carefully considered and more importance must be given to it in the future than has in times past.

The cementum is a separate calcified tissue developing specialized mesoblastic cells covering the dentin of the tooth and binding the ends of the peridental membrane into the cementum, thereby forming a means of attachment between the dentin and the peridental membrane.

When the separate and distinct function of each of these three tissues is considered, and that each one to a certain extent is independent histologically of the other from the developmental standpoint, much clearer clinical knowledge can be obtained than has been in the past when the peridental membrane was considered to be the source of development for both the osteoblasts and cementoblasts. —*Journal National Dental Association.*

